# Antiviral Drug Candidate Repositioning for *Streptococcus suis* Infection in Non-Tumorigenic Cell Models

**DOI:** 10.3390/biomedicines12040783

**Published:** 2024-04-02

**Authors:** Ashley Anzet van Niekerk, Sara Maluck, Patrik Mag, Csaba Kővágó, Ádám Kerek, Ákos Jerzsele, Torsten Steinmetzer, Erzsébet Pászti-Gere

**Affiliations:** 1 Department of Pharmacology and Toxicology, University of Veterinary Medicine, István utca 2, H-1078 Budapest, Hungaryjerzsele.akos@univet.hu (Á.J.); 2 National Laboratory of Infectious Animal Diseases, Antimicrobial Resistance, Veterinary Public Health and Food Chain Safety, University of Veterinary Medicine, István utca 2, H-1078 Budapest, Hungary; 3 Faculty of Pharmacy, Institute of Pharmaceutical Chemistry, Philipps University Marburg, Marbacher Weg 6, 35032 Marburg, Germany

**Keywords:** IPEC-J2 cells, *Streptococcus suis*, oxidative stress, hepatocytes, cytotoxicity, 3-amidinophenyalanine

## Abstract

The increasing prevalence of antimicrobial resistance against zoonotic bacteria, including *Streptococcus (S.) suis*, highlights the need for new therapeutical strategies, including the repositioning of drugs. In this study, susceptibilities of bacterial isolates were tested toward ten different 3-amidinophenyalanine (Phe(3-Am)) derivatives via determination of minimum inhibitory concentration (MIC) values. Some of these protease inhibitors, like compounds MI-432, MI-471, and MI-476, showed excellent antibacterial effects against *S. suis*. Their drug interaction potential was investigated using human liver microsomal cytochrome P450 (CYP450) measurements. In our work, non-tumorigenic IPEC-J2 cells and primary porcine hepatocytes were infected with *S. suis*, and the putative beneficial impact of these inhibitors was investigated on cell viability (Neutral red assay), on interleukin (IL)-6 levels (ELISA technique), and on redox balance (Amplex red method). The antibacterial inhibitors prevented *S. suis*-induced cell death (except MI-432) and decreased proinflammatory IL-6 levels. It was also found that MI-432 and MI-476 had antioxidant effects in an intestinal cell model upon *S. suis* infection. Concentration-dependent suppression of CYP3A4 function was found via application of all three inhibitors. In conclusion, our study suggests that the potential antiviral Phe(3-Am) derivatives with 2′,4′ dichloro-biphenyl moieties can be considered as effective drug candidates against *S. suis* infection due to their antibacterial effects.

## 1. Introduction

In large-scale pig farming, the post-weaning period is most crucial as piglets are more susceptible to environmental pathogens due to an impaired immune system [1]. *Streptococcus suis* (*S. suis*) is one of the most important bacterial species causing damage to swine health and, thus, significant economic loss worldwide [2,3]. Due to its zoonotic potential, it is still a serious public health concern in many countries [4]. A total of 35 serotypes of *S. suis* (1–34 and 1/2) were originally classified based on the antigenicity of the capsular polysaccharide (CPS) [2,5], but recent studies have shown that 6 of these serotypes (20, 22, 26, and 32–34) belong to different bacterial species [6,7]. Serotype 2 is most frequently responsible for the development of clinical signs in both humans and pigs [8]. The bacteria colonize the upper respiratory tract (tonsil, nasopharynx) after birth and become part of the normal microflora [9]. As a secondary pathogen, it is involved in the porcine respiratory disease complex due to predisposing factors [10] and, by entering the bloodstream, it can lead to pneumonia, septicemia, arthritis, and meningitis, the latter with high mortality [11].

While in Europe and America, human infections are caused by injuries [8], in Asia, the disease is caused by the consumption of swine meat following inadequate heat treatment [12]. Therefore, it has been suggested that in addition to oronasal transmission, intestinal translocation may also play an important role in the spread of infection [13]. Human symptoms also include meningitis, arthritis, and sepsis [14,15], and endocarditis has been described too [16].

With regard to epithelial damage, *S. suis* serotypes 2 and 9 possess differently composed CPS, which impacts bacterial adhesion to human colorectal adenocarcinoma cells (Caco-2) or intestinal porcine epithelial cells-jejunum 2 (IPEC-J2) cells via modulating the binding affinity of bacteria to host intestinal epithelial cells. Additionally, other variants of streptococci have been proven to translocate across human Caco-2 cells via the paracellular route, which is a common route for bacterial pathogens in epithelial cells to penetrate intestinal mucosa [17]. Other organs, such as the liver, spleen, kidney, or heart, were also observed to be invaded as *S. suis* enters the systemic circulation after bacterial penetration of host mucosal barriers and survival in the blood cells [18,19].

Bacterial infections in pigs are often treated with antibiotics; however, inappropriate usage of these substances leads to the emergence of resistant strains. During an infection caused by *S. suis*, β-lactams are mostly effective in eliminating the pathogen. In recent studies, including the European VetPath survey between 2005 and 2012 [20], 100% and 97% of *S. suis* strains isolated from pigs were found to be sensitive to amoxicillin–clavulanic acid and ceftiofur, respectively. The low minimum inhibitory concentration values for amoxicillin and cefquinome also indicate a high susceptibility of the bacterium. In the same survey, more than 95% of strains were sensitive to enrofloxacin and florfenicol, but high resistance was detected to tetracycline (only 4% of strains were sensitive) [21]. The β-lactams, enrofloxacin, and florfenicol remain effective against the majority of *S. suis* infections, based on monitoring programs in several European countries [22,23]; however, there is increasing concern about the detection of fluoroquinolone- and florfenicol-resistant strains in some areas of Asia [3,24]. In a recent study, susceptibility testing of *S. suis* strains obtained in different locations in Thailand displayed widespread resistance to tetracyclines and macrolides. *S. suis* obtained from asymptomatic pigs showed only intermediate susceptibility to gentamicin, penicillin, norfloxacin, and enrofloxacin, indicating an onset of *S. suis* antibiotic resistance [25].

The antiviral effects of 3-amidinophenyalanine (Phe(3-Am))-derived inhibitors of host proteases such as matriptase and transmembrane serine protease 2 (TMPRSS2) are based on the inhibition of viral fusion with the host cell, which is essential for the replication and spread of certain influenza and coronaviruses. Host proteases located in the TGN and/or on the cell surface are responsible for the proteolytic cleavage of the influenza haemagglutinin precursor (HA0) of influenza virus (H1N1 and H7N9) and spike protein (S) of Middle East respiratory syndrome coronavirus (MERS-CoV) and severe acute respiratory syndrome coronavirus (SARS-CoV) and SARS-CoV-2 [26,27,28,29,30,31,32]. The Phe(3-Am)-derived matriptase/TMPRSS2 inhibitors MI-432 and MI-1900 have already been found to be effective against SARS-CoV-2 in human airway cells synergistically in combination with the furin inhibitor MI-1851 [30].

Due to the development of antimicrobial resistances (AMRs), there is an increasing need to discover alternative solutions for replacing antibiotic treatment, which includes vaccination of sows in the last trimester of pregnancy. It is also possible to produce herd-specific vaccines. Another alternative option is the repositioning of already authorized agents and novel drug candidates for various therapeutic indications instead of developing new antibacterial agents from scratch. Serine, cysteine, and metalloproteases are prevalent across various pathogenic bacteria. They play a vital function in colonization, attacking host immune responses, relying on host nutrients for growth, and inducing tissue damage during infection. Presently, most of the therapeutically used antibiotics primarily target bacterial cell wall biosynthesis or interfere with protein synthesis on ribosomes. However, the widespread resistance to these antibiotics poses a significant medical challenge. Therefore, there is an increasing demand for novel or repurposed drugs with diverse mechanisms of action, leading to the emergence of a new generation of antibiotics that specifically target bacterial proteolytic enzymes and consequently offer potential solutions to combat antibiotic resistance effectively. Development of these protease blockers appears to be a promising approach to address AMR-related ineffective drug therapy [33,34,35].

In our study, MICs of ten Phe(3-Am) derivatives were determined against eight bacterial strains. To assess drug interaction potential of the inhibitors, changes in activities of liver microsomal cytochrome P 450 (CYP) isoenzymes such as CYP1A2, CYP2C9, CYP2C19, CYP2D6, and CYP3D4 were measured. The cytotoxic effects of *S. suis* alone and the presence of protease inhibitors with antibacterial efficacy were determined by measuring the viability of IPEC-J2 cells and primary porcine hepatocytes. Additionally, hydrogen peroxide (H_2_O_2_) production was monitored extracellularly (EC) after 24 h exposure to IPEC-J2 cells or swine hepatocytes to *S. suis* alone or with inhibitors. To detect cellular changes in inflammatory processes induced by *S. suis* administration, proinflammatory interleukin (IL)-6 levels were measured in the presence of inhibitors.

## 2. Materials and Methods

### 2.1. Chemicals

All chemicals were purchased from Merck (Darmstadt, Germany), including, dimethyl sulfoxide (DMSO), Neutral Red (NR) dye, IL-6 porcine ELISA, and an Amplex red hydrogen peroxide assay kit. Mueller–Hinton liquid broth was procured from Biolab Ltd. (Budapest, Hungary). The Phe(3-Am) derivatives were prepared at the Faculty of Pharmacy, Institute of Pharmaceutical Chemistry, Philipps University Marburg, Germany.

### 2.2. Preparation of Inhibitor Solutions for IPEC-J2, Microsomal, and Hepatocyte Assays

**Figure 1:**
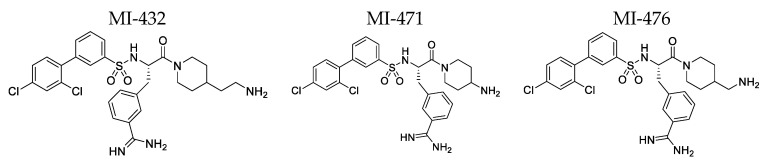
Chemical structures of the applied inhibitors.

The chemical structures of the protease inhibitors MI-432, MI-471, and MI-476 are shown in Figure 1, and the structures of the other seven tested Phe(3-Am) derivatives are provided in Figure 1 and Figure S1. A total of 10 mM of stock solutions in DMSO were prepared and kept at −20 °C. Freshly made working solutions for the inhibitors were prepared prior to each study. Following incubation of the IPEC-J2 cells, hepatocytes, or microsomes with the inhibitors at 37 °C in a humidified atmosphere of 5% CO_2_, the solutions underwent subsequent spectrophotometric (NR, IL-6 ELISA), fluorometric (CYP assays, Amplex red method), or UPLC/MS-MS procedures (depletion %).

### 2.3. MIC Measurements

Bacterial strains used in this study were as follows: *S. suis* serotype 2 (ATCC 958242) *Staphylococcus (S.) aureus* (ATCC 10832), *Escherichia (E.) coli* (ATCC 11775), *Pseudomonas (P.) aeruginosa* (ATCC 27853), *Pasteurella (P.) multocida* (clinical isolate), *Burkholderia (B.) cepacia* (clinical isolate), *Salmonella (S.) enterica* (clinical isolate), and *Enterococcus (E.) faecalis* (clinical isolate). Of the clinical isolates used in this study, *P. multocida* is of porcine origin, B. cepacia is of human origin, and *S. enterica* and *E. faecalis* are of poultry origin. The bacterial strains were stored in the Microbiology Laboratory of the University of Veterinary Medicine, Budapest, at a temperature of −80 °C. Bacterial strains were inoculated the day before the test in 5 mL of Cation-Adjusted Mueller–Hinton Broth (CAMHB) and incubated for 18–24 h at 37 °C. The initial inoculum concentration used for the MIC assay was 5 × 10^5^/mL. All wells except the first column of the working plates were filled with 90 µL CAMHB. A stock solution of inhibitors at 800 µM was then prepared, 180 µL of which was measured into the first column of plates and used to prepare a 2-based dilution on 96-well plates. After the 10th column, the tips were discarded with the 90 µL excess solution so that each column contained 90 µL of solution. Each bacterial strain was tested in a single row on the working plates. A bacterial suspension diluted to 25× was prepared on an auxiliary plate for bacterial inoculation by loading 240 µL of TSB per column of plates and adding 10–10 µL of bacterial strain suspension per well. In the next step, the isolated bacterial strains were inoculated onto the plates. Starting from column 11 (positive control) of the plates containing dilution row 2, 10 µL of bacterial suspension was added to each well, working backward. Column 11 served as a positive control (containing only bacterial suspension and broth), while column 12 served as a negative control (containing only broth). The microplates were then incubated at 37 °C for 18–24 h, and the MIC values were assessed in comparison to positive controls.

### 2.4. IPEC-J2 Cell Culture and Cytotoxicity Assay

The IPEC-J2 cell line is unique as it is non-transformed and non-tumorigenic. IPEC-J2 cells are derived from intestinal enterocytes extracted from the jejunum of neonatal unsuckled piglets [36]. IPEC-J2 cells were gifted by Dr. Jody Gookin (Department of Clinical Sciences, College of Veterinary Medicine, North Carolina State University, Raleigh, NC, USA). The cells were propagated in a medium containing Dulbecco’s modified Eagle’s medium (DMEM) and Ham’s F-12 Nutrient (1:1, DMEM/F12), supplemented with 1% insulin/selenium/transferrin, 5 ng/mL epidermal growth factor (EGF), 5% fetal bovine serum, and 1% penicillin–streptomycin. The cells had undergone passage around 50 times. The potential cytotoxic effects of *S. suis* (10^4^ CFU/mL), inhibitors (at 50 µM), and their combinations were evaluated on IPEC-J2 cells seeded on 96-well plates using the 2 h lasting NR method [37]. This experiment included plain DMEM/F12 medium as a control. At the end of the final treatments (24 h), the ratio of viable cells was measured by absorbance at 540 nm wavelength with a SpectraMax iD3 microplate reader.

### 2.5. Cytotoxicity Assays in Hepatocytes

Porcine cryopreserved primary hepatocytes were obtained from Primacyt Cell Culture Company (Schwerin, Germany). Lonza Group Ltd. (Biocenter Ltd., Szeged, Hungary) supplied the thawing, plating, and maintenance media. The cytotoxic effects of inhibitors MI-432, MI-471, and MI-476 were tested on the viability of primary hepatocytes using the NR method. The hepatocytes were cultivated on a 96-well plate for 24 h, followed by incubation with the inhibitors at 0, 50, and 100 µM for an additional 24 h. Only cell maintenance medium was used to incubate the control cells. The plate underwent incubation together with NR dye for 2 h. Hepatocyte viability was recorded at 540 nm with a SpectraMax iD3 microplate reader.

### 2.6. CYP Enzyme Fluorometric Activity Measurements

The Biovision CYP assays (BioVision, Inc., Kampenhout, Belgium) employ non-fluorescent CYP2D6, 2C9, 2C19, 1A2, or 3A4 substrates able to undergo transformation into highly fluorescent detectable metabolites. In these experiments, positive controls such as quinidine (CYP2D6, 3 µM), tienilic acid (CYP2C9, 60 µM), (+)-N-3-benzylnirvanol (CYP2C19, 30 µM), α-naphthoflavone (CYP1A2, 6 µM), and ketoconazole (CYP3A4, 5 µM) were selected. Human hepatic microsomal supernatants were prepared (Gibco, Biocentre, Szeged, Hungary protein concentration: 20 mg/mL) separately by mixing 30 µL with 2425 µL assay buffer and with 50 µL nicotinamide adenine dinucleotide phosphate (NADPH) generating system (100×). A total of 20 µL of the protease inhibitor solutions (250 µM for all CYP enzymes except CYP3A4, where 50, 125, and 250 µM have been used) were added to aliquots of 50 µL microsomal suspensions. Control measurements were performed in the absence of the inhibitors.

In negative controls, 20 µL assay buffer and in positive controls, the reference inhibitors (each 20 µL; α-naphthoflavone, 30 µM, tienilic acid, 300 µM; (+)- N-3-benzylnirvanol, 150 µM; ketoconazole, 150 µM, quinidine, 15 µM) were added to the 50 µL microsome-containing buffer. In background controls, only assay buffer excluding microsomes and test compounds was used in a volume of 70 µL. Subsequently, after incubation at 37 °C for 15 min, 30 µL of the suitable CYP substrate/NADP+ mixture was incorporated into each well, resulting in a final reaction volume of 100 µL/well. The obtained microsomal protein content was assessed using the bicinchoninic acid protein assay kit (Pierce BCA kit, Thermo Fisher Scientific, Waltham, MA, USA). In each assay, the microsomal protein concentration was calibrated to 100 µg/well. The fluorescence intensities were measured with a fluorometer (Victor X2 2030, Perkin Elmer, Waltham, MA, USA) using λex/em =  406/468 nm for CYP1A2 and for CYP2C19, λex/em =  415/502 nm for CYP2C9, λex/em =  390/468 nm for CYP2D6, and λex/em =  535/587 nm for CYP3A4 assays. The solvents used (≤0.5%DMSO and ≤1% acetonitrile (ACN)) on the function of the tested CYP enzymes did not cause significant inhibition.

### 2.7. Examination of EC H₂O₂ Status

For oxidative status measurements, inhibitors MI-432, MI-471, or MI-476 were used at 50 µM in the absence and presence of *S. suis* for 24 h in IPEC-J2 cells and in primary porcine hepatocytes on 24-well plates. An Amplex red hydrogen peroxide assay kit was used to monitor EC hydrogen peroxide (H₂O₂) production in IPEC-J2 cells and in hepatocytes. Following the manufacturer’s instructions, cell-free supernatants were collected and mixed with the Amplex red working solution (in a ratio of 1:1). A Victor X2 2030 fluorometer was used to detect fluorescence intensity at wavelengths 530 nm and 590 nm for excitation and emission, respectively.

### 2.8. Evaluation of Proinflammatory Cytokine IL-6 Expression

After 24 h incubation time, the cell-free supernatants from MI-431, MI-471, and MI-476 exposure were sampled from IPEC-J2 cells and from porcine hepatocytes. The changes in cytokine levels were detected with porcine IL-6 ELISA kits. The manufacturer’s instructions were followed to treat the supernatants and measured by a SpectraMax iD3 microplate reader at 450 nm.

### 2.9. Statistical Analysis

The statistical evaluation of the data was executed using version 2023 R Core Team. One-way ANOVA was used to determine differences between groups. Post hoc Tukey was applied for multiple comparisons. *p* < 0.05, *p* < 0.01, and *p* < 0.001 indicate statistically significant differences.

## 3. Results

### 3.1. Determination of MIC Values of Inhibitors

The test substances (Figure 1 and Figure S1) were not able to inhibit bacterial growth against *P. aeruginosa*, *E. coli*, *S. enterica*, and *E. faecalis* (MIC > 800 µM). Only test substances 471 and 476 were able to inhibit the growth of *B. cepacia* isolates to a moderate extent (MIC at 400 µM). Against the *S. aureus* clinical isolate, test substance 432 showed a significant inhibitory effect at MIC values of 50 µM, whereas test substances 471, 476, and 485 only moderately inhibited bacterial growth (MIC 100–200 µM), while the other tested substances had no significant effect on bacterial growth. Inhibitors 432, 471, and 476 had a significant inhibitory effect on *P. multocida* clinical isolates at MIC values of 50 µM, while test substances 472 and 477 only moderately inhibited bacterial growth (MIC at 400 µM), and the other test substances did not significantly affect bacterial growth. The highest efficacy of the screened inhibitors was observed against *S. suis*, where test substances 432, 471, and 476 inhibited bacterial growth at 1.5 µM, 3 µM, and 6 µM, respectively. The results are summarized in Table 1.

### 3.2. Influence on Human CYP3A4, 1A2, 2D6, 2C9, and 2C19 Activities

**Figure 2:**
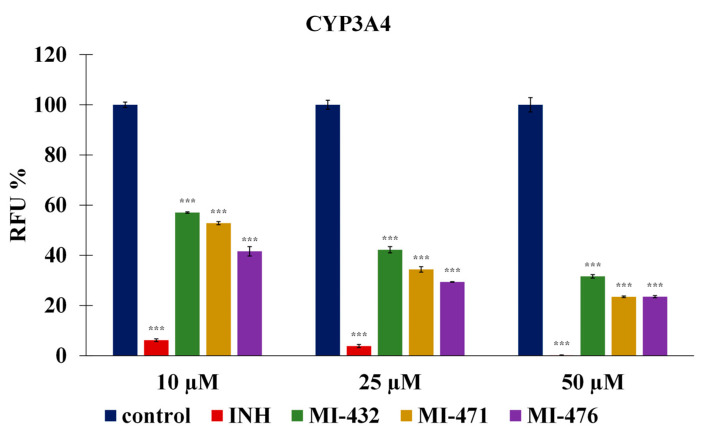
The effect of the protease inhibitors at 10 µM, 25 µM, and 50 µM on human hepatic microsomal CYP3A4 isoenzyme function. The microsomal preparations were treated by the inhibitors for 15 min at 37 °C. The reference inhibitor ketoconazole (INH) was used at a concentration of 5 µM and significantly suppressed CYP3A4 activities in humans (*** *p* < 0.001). The expressed data are the mean relative fluorescence intensities (RFUs) shown as a percentage of the untreated control fluorescence values ± SD (n = 3); *** signifies *p* < 0.001 compared to controls.

The following experiments have been only conducted with the three inhibitors MI-432, -471, and -476, which showed the highest antibacterial effects against *S. suis*. Inhibitor concentrations of 10 µM, 25 µM, and 50 µM were added to human microsomal preparations for 15 min, using ketoconazole as a CYP3A4 reference inhibitor. It was observed that CYP3A4 isoenzyme activities were mitigated by the addition of inhibitors at 10 µM (*p* < 0.0001), 25 µM (*p* < 0.0001), and 50 µM (*p* < 0.0001), as seen in Figure 2.

**Figure 3:**
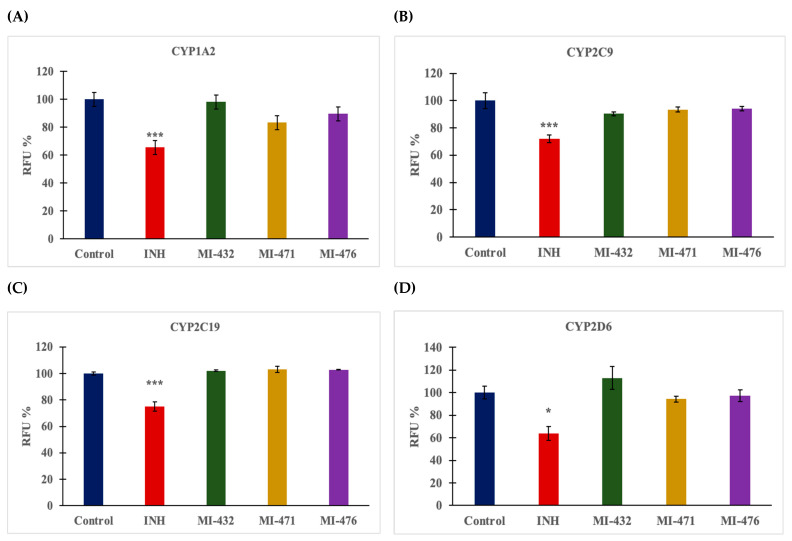
The effect of the protease inhibitors at 50 µM on human hepatic microsomal CYP1A2 (**A**), 2D6 (**B**), 2C9 (**C**), and 2C19 (**D**) isoenzyme activities. The microsomal preparations were treated by the inhibitors for 15 min at 37 °C. The reference inhibitor α-naphthoflavone (α-NF) used at a concentration of 6 µM significantly suppressed CYP1A2 activities in humans (*** *p* < 0.001); however, neither of the protease inhibitors at 50 µM changed CYP1A2 activity. The reference inhibitor, quinidine, used at 3 µM, significantly suppressed CYP2D6 activities (* *p* < 0.05). Furthermore, the reference inhibitors tienilic acid (TA) at 60 µM and (+)-N-3 benzylnirvanol (BN) at 30 µM significantly suppressed CYP2C9 and CYP2C19 function (*** *p* < 0.001). The expressed data are the mean relative fluorescence intensities (RFUs) shown as a percentage of the untreated control fluorescence values ± SD (n = 3–4); * signifies *p* < 0.05, and *** signifies *p* < 0.001 compared to controls.

Similar results are shown in Figure 3, where the same inhibitors were added at 50 µM to human microsomal preparations for 15 min using α-naphthoflavone, quinidine, tienilic acid, and N-3-benzylnirvanol as reference inhibitors for measurements with CYP1A, 2D6, 2C9, and 2C19, respectively. Together, all four CYP isoenzyme activities were not mitigated by the addition of these inhibitors (*p* > 0.05).

### 3.3. Cell Viability Assay

**Figure 4:**
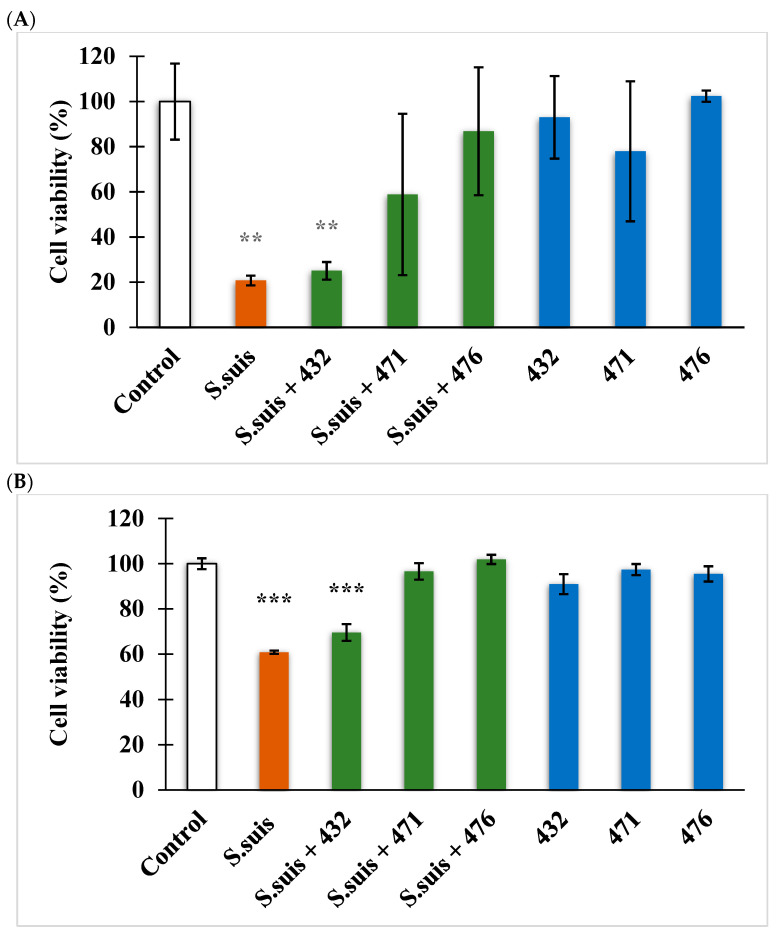
Determination of cell viability in IPEC-J2 cells (**A**) and swine hepatocytes (**B**). Changes in cell viability determined by absorbance measurements after 24 h of administration of 10^4^ CFU/mL *S. suis*, (MI-)432, (MI-)471, and (MI-)476 at 50 µM and their combinations. Data are represented as cell viability % expressed in control % with standard errors of mean (SEM), n = 3–4 samples per group. ** indicates *p* < 0.01 and *** signifies *p* < 0.001 compared to controls.

Prior to 2 h of incubation with Neutral red dye, the viability of IPEC-J2 cells exposed to *S. suis* for 24 h in the absence or in the presence of inhibitors was measured (Figure 4A). *S. suis* (10^4^ CFU/mL) increased the death rate of the cells (*p* = 0.00248), and in combination with inhibitor MI-432 (50 µM), a slightly less worsening of the cells can be seen (*p* = 0.00436). However, *S. suis* did not show any negative effect on cell viability together with inhibitors MI-471 and MI-476 at 50 µM after 24 h treatment (*p* > 0.05). It was also ascertained that all three inhibitors alone at 50 µM for 24 h did not cause significant cell death (*p* > 0.05). It was also seen in Figure 4B that *S.suis* significantly decreased cell viability in porcine hepatocytes (*p* < 0.001) even in combination with inhibitor MI-432 (*p* < 0.001). In contrast, *S.suis* did not deteriorate cell viability in primary porcine hepatocytes when combined with inhibitors MI-471 and MI-476 at 50 µM (*p* > 0.05). It was also discovered that all three inhibitors alone at 50 µM for 24 h did not result in significant damage to porcine hepatocytes (*p* > 0.05).

### 3.4. Determination of EC ROS Status

**Figure 5:**
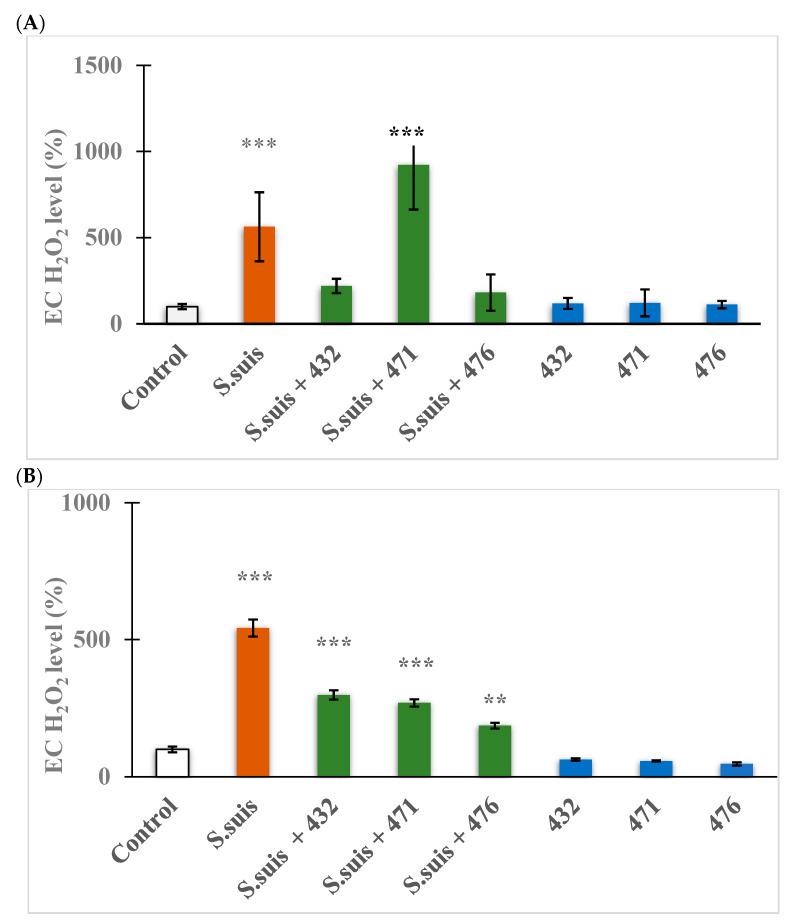
Measurement of EC H₂O₂ production after treatment with *S. suis* (10^4^ CFU/mL) alone or in combination with the indicated inhibitors (MI-)432, (MI-)471, and (MI-)476 at a concentration of 50 µM in IPEC-J2 cells (**A**) and in hepatocytes (**B**) using the Amplex red method. The EC H_2_O_2_ levels were determined by measurements of the relative fluorescence intensities compared to the control (100%) with standard errors of mean (SEM), n = 3 samples per group. ** signifies *p* < 0.01, and *** signifies *p* < 0.001 compared to controls.

A significant increase in EC H₂O₂ secretion in IPEC-J2 cells exposed to *S. suis* (10^4^ CFU/mL) alone or in combination with inhibitor MI-471 (*** *p* < 0.001) can be seen in Figure 5A. However, IPEC-J2 treated with *S. suis* together with inhibitors MI-432 and MI-476 at 50 µM after 24 h treatment had unchanged H₂O₂ production (*p* > 0.05), similar to inhibitors MI-432, MI-471, or MI-476 alone (*p* > 0.05). In Figure 5B, hepatocytes treated with *S. suis* (10^4^ CFU/mL) alone and in combination with all inhibitors significantly elevated EC H₂O₂ levels (** *p* < 0.01 in each treatment). However, hepatocytes treated with the inhibitors alone showed no change in redox balance (*p* > 0.05).

### 3.5. Changes in Proinflammatory Cytokine IL-6 Expression

**Figure 6:**
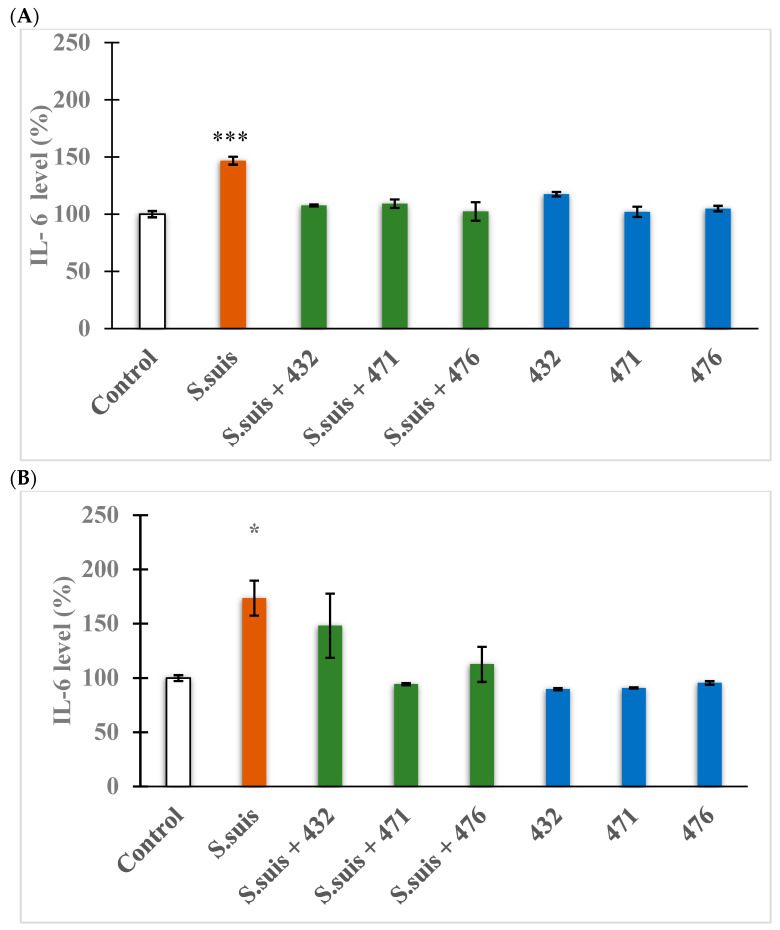
Measurement of interleukin-6 production after 24 h treatment with *S. suis* (10^4^ CFU/mL) alone or in combination with the indicated inhibitors (MI-)432, (MI-)471, and (MI-)476 or with the inhibitors alone in IPEC-J2 cells (**A**) and in hepatocytes (**B**). Data are represented as IL-6 levels % expressed in control with standard errors of mean (SEM), n = 3–4 samples per group. * signifies *p* < 0.05 and *** indicates *p* < 0.001 compared to controls.

Figure 6A shows an elevated IL-6 production in cell-free supernatants of IPEC-J2 cells exposed to *S. suis* (10^4^ CFU/mL) alone (*** *p* < 0.001). All inhibitors alone and in combination with *S. suis* for 24 h did not significantly raise IL-6 levels (*p* > 0.05). Similarly, as seen in Figure 6B, IL-6 production was increased in hepatocytes to a greater extent after exposure to *S. suis* alone (* *p* < 0.05). In contrast, no elevation was seen in IL-6 levels in hepatocytes after exposure to *S. suis* in combination with all three inhibitors (*p* > 0.05) or with the inhibitors alone (*p* > 0.05).

## 4. Discussion

*S. suis* is a worldwide zoonotic pathogen and still poses a major public health risk in many countries [3,11]. Treatment is primarily based on β-lactams, which have been shown to be still effective [21]. However, there is concern about the increasing emergence of strains resistant to florfenicol and enrofloxacin, an AMEG B category antibiotic, due to inappropriate antibiotic use [3,24,25] in some countries. Among the bacterial isolates screened, *P. aeruginosa*, *E. coli*, *S. enterica*, and *E. faecalis* could not be inhibited by the test substances, even at very high, nearly millimolar concentrations. In the case of *B. cepacia*, a minor antibacterial activity was only observed at high concentrations (400 µM) of the test substances MI-471, MI-476, and MI-1904, and against *P. multocida* and *S. aureus*, some inhibitory activity was found at lower concentrations (50 µM) for several test substances. The most pronounced suppression was obtained with six test compounds against *S. suis*, which showed antibacterial activity even at concentrations of 50 µM or lower. The lowest MICs were found in the presence of agents MI-432, MI-471, and MI-476. However, further in vivo studies are needed to evaluate the antibacterial effect in animal models.

All compounds that are currently examined can be described as sulfonylated tertiary amides of the unnatural amino acid Phe(3-Am). The N terminus of the Phe(3-Am) is coupled with a 2′,4′ disubstituted (either dichloro- or dimethoxy) 3-sulphonyl-biphenyl moiety, while its carboxyl group is amidated with various piperidine derivatives containing either a terminal free amino or an ureido group. The benzamidine moiety in the side chain of Phe(3-Am) is a strongly basic group, and the best antibacterial effects were found for dibasic compounds, which contain an additional amino group on the piperidine moiety. In contrast, a negligible antibacterial effect was observed for compound MI-490, although it is also a dibasic inhibitor containing its second basic group in a different position at the N-terminal biphenyl moiety. Furthermore, all of the most effective compounds (MIC values against *S. suis*: MI-432, <1.5625 µM; MI-471, 3.125 µM; MI-476, 6.25 µM) contain an N-terminal 2′,4′ disubstituted biphenyl moiety. Notably, the 2′,4′-dichloro-substituted compounds have a stronger antibacterial activity compared with the otherwise structurally similar 2′,4′-dimethoxy-substituted derivatives, and the latter compounds had to be used at significantly larger concentrations. In addition, no measurable antibacterial effect against any of the tested strains was found for the monobasic compound MI-463, which contains a very bulky cyclohexyl-substituted C-terminal ureido group.

The inhibitory effects on CYP activities were determined in human liver microsomes to assess the potential drug interaction of the applied inhibitors. No significant inhibitory effects of compounds MI-432, MI-471, and MI-476 were found on CYP1A2, CYP2C9, CYP2C19, and CYP2D6 activities. In contrast, a previous study has shown a significant inhibition of monkey CYP1A2 enzymes after the administration of inhibitor MI-432 [38], suggesting species-dependent differences in the CYP-modulating function of the compounds. Otherwise, inhibitors MI-432, MI-471, and MI-476 significantly mitigated CYP3A4 activity corresponding to previous findings, whereas other Phe(3-Am) derivatives demonstrated similar inhibitory effects on CYP3A4 function [39].

IPEC-J2 cells have been proven to be an appropriate model for investigating intestinal functions. The suitability of IPEC-J2 cells is based on their sufficient properties for bacterial adhesion and cellular entrance [40]. Not only are these features present, but the superiority of IPEC-J2 cells in this study over other typically used intestinal cell lines such as HT-29, Caco-2, or T84 can also be explained by considering their carcinogenicity. These cell lines, which are derived from different types of carcinomas found in the colon, may have characteristics that are non-physiological. The relative lack of responsiveness to cytokines, as well as the adapted glycosylation pattern or deviating protein expression, lead to the preferred use of IPEC-J2 over the cancerous cell types [41]. The primary porcine hepatocytes used in this study better mimic physiological in vivo conditions because they are not derived from a hepatoma, as is the case with commonly used cell lines such as HepG2. Although human liver cancer cells have some benefits, like a long lifespan and a stable phenotype, they are not reliable for most drug-metabolizing enzymes and hepatotoxicity studies due to their limited expression [42].

Examining the effect of *S. suis* on IPEC-J2 is important not only in the veterinary field to improve gut health in pigs for higher production rates but also because of its similarity to the human epithelium, which provides insight into its effect on the human gut [43]. The porcine IPEC-J2 cell line has been widely used in microbiological experiments to model the interactions between various pathogens and the jejunum. Several cell studies involving the infection of intestinal epithelial cells with Enterobacteriaceae such as *S. enterica* and pathogenic *E. coli* have been published recently [44,45]. Inflammatory cytokine IL-8 and tumor necrosis factor (TNF)-α levels of porcine enterocytes treated with Lawsonia intracellularis alone or in the presence of *S. enterica* serovar Typhimurium were elevated significantly, and synergistic effects were observed in case of co-infection [46].

In our studies, IPEC-J2 and primary pig hepatocytes were used as cell-based models to study the (sub)cellular changes induced by *S. suis* infection and to monitor the potential beneficial effects of the repurposed antiviral drug candidates. To the best of our knowledge, this study was the first to measure the effect of this type of matriptase/TMPRSS2 inhibitor on the cell viability and redox status of IPEC-J2 cells and primary porcine hepatocytes in the presence of *S. suis*. It was shown that treatment with *S. suis* at 10^4^ CFU/mL for 24 h had a negative effect on the cell viability of IPEC-J2 cells and hepatocytes. In the cell viability assay, a reduction in the number of viable cells in both IPEC-J2 cells and hepatocytes was also observed after treatment with *S. suis* in combination with inhibitor MI-432. In contrast, the cytotoxic impact of *S. suis* was effectively suppressed via concomitant administration of compounds MI-471 and MI-476 in both cell models.

Previous studies have shown deviations in certain interleukin levels after treatment with Enterobacteriaceae. However, all proinflammatory cytokines can be indicators of inflammatory processes if they are elevated above physiological values. The focus of this study was on the measurement of IL-6 levels, which are primarily responsible for inducing acute phase proteins [47]. Both IPEC-J2 and primary pig hepatocytes are capable of cytokine production [40,44,47], and they showed significant increases in IL-6 levels after *S. suis* treatment after 24 h. Interestingly, together with an inhibitor treatment, the IL-6 levels were no longer significantly increased, which is beneficial because elevated IL-6 serum levels can be considered as a precursor for serious hepatic diseases, such as hepatocellular carcinoma [47]. In summary, significant anti-inflammatory effects were found in cell-based models challenged with *S. suis* upon administration of MI-432, MI-471, and MI-476.

The two cell lines used in this study are also suitable for measuring oxidative stress levels since they are both exposed to high levels of reactive oxygen species (ROS) under physiological conditions. Issues arise when the ROS exceeds antioxidant capacity, called oxidative stress, and processes such as lipid peroxidation or protein damage occur, ultimately leading to cell death [48,49]. The IPEC-J2 experiment revealed a significant elevation in H_2_O_2_ levels when *S. suis* was used alone and when bacterial exposure was combined with MI-471. This phenomenon requires further investigation. In contrast, compounds MI-432 and MI-476 could exert antioxidant properties in *S. suis* infection. The hepatocytes exhibited significantly increased H_2_O_2_ values when infected with *S. suis*, which could not be reduced significantly by adding the inhibitors. Treatment with the inhibitors alone did not result in an elevated H_2_O_2_ level, indicating that they can be safely used alone without causing redox imbalance.

Inhibition of the proteolytic cleavage of the surface glycoprotein haemagglutinin prevents the influenza virus from entering the cell, thereby preventing replication and infection [30,31]. Even though this concept has mainly been optimized in the context of influenza virus replication inhibition, applying this mechanism to SARS-CoV-2 could offer a potential treatment strategy against COVID-19. It is essential to inhibit the proteolytic cleavage at the S2′ site of the spike protein, which is a substrate for TMPRSS2 and is located on the surface of the virus itself. TMPRSS2 appears to be the most important transmembrane serine protease associated with coronavirus replication. By preventing the direct fusion of the virus with the host cell membrane, the inhibitor would provide concentration-dependent inhibition of virus replication. So far, these mechanisms have been successfully demonstrated mainly in Calu-3 cells [30,31].

Proteolytic enzymatic activities have been considered important virulence factors in host–pathogen interaction. Several types of proteases were identified and characterized as produced by *S. suis,* including cell-associated and EC Arg-aminopeptidase, chymotrypsin-like and caseinase activities of serine- and metalloprotease classes, and DPP IV [50]. Recently, there have been emerging findings that cysteine protease ApdS from *S. suis* takes part in the cleavage of the antimicrobial peptide cathelicidin LL-37; thus, bacteria can evade LL-37-triggered immune processes [51]. In addition, a novel specific complement evasion factor, prokaryotic immunoglobulin M protease in *S. suis,* is also involved in adaptive immune response [52]. Until now, the antibacterial effects of Phe(3-Am) derivatives have not been described in *S. suis*-caused infection. Only structurally similar synthetic inhibitors of benzamidine type were proven to have inhibitory effects on cysteine proteinases on the virulence of *Porphyromonas gingivalis* strains [53]. Further studies should, however, be conducted to determine the modulatory function of the applied Phe(3-Am) inhibitors on certain proteases necessary for bacterial invasion.

The concept of drug repurposing, also known as drug repositioning, is a special area of research that aims to develop specific drugs for new or variations of diseases in a faster manner. Shortening the duration of development and reducing costs for repurposed drugs is also facilitated by the presence of basic knowledge about the potential adverse effects of these compounds. Other examples include drugs that have not reached the market yet for their intended purpose but have been found to possess additional functions that enable their use against other targets. On this basis, the potential for drug repurposing is extensive [54,55]. In this study, the antibacterial effects of three inhibitors against matriptase and/or TMPRSS2 were confirmed against *S. suis* at low micromolar concentrations. A drug repurposing of already existing serine protease inhibitors could be a suitable strategy to speed up the process of providing sufficient treatment options against resistant bacterial strains instead of overusing critically important antibiotics.

## 5. Conclusions

Due to the zoonotic potential of *S. suis* and the development of AMR because of frequent antibiotics usage, the repurposing of authorized drugs and drug candidates has been of key interest worldwide. In our work, several Phe(3-Am)-derived matriptase/TMPRSS2 inhibitors were screened for their antibacterial properties in vitro. It was demonstrated that inhibitors MI-471 and MI-476 possess a significant antibacterial efficacy against *S. suis* infections without affecting the cell viability and exhibiting anti-inflammatory properties. For inhibitors MI-432 and MI-476, an antioxidant effect in intestinal epithelial cells upon *S. suis* infection was found. Conductance of in vivo studies in the future could further reflect the efficacy and potency of the antimicrobial Phe(3-Am) derivatives with 2′,4′ dichloro-biphenyl moieties against *S. suis* infection.

## Figures and Tables

**Table 1:** Efficacy of the tested substances against different bacterial isolates. The green color indicates sensitive isolates, the yellow color shows moderately sensitive isolates, and the red color refers to isolates resistant to each applied inhibitor.

Strains	Sources	MIC (µM) of Phe(3-Am)-Derived Protease Inhibitors									
		432	463	471	472	476	477	485	490	1903	1904
*P. aeruginosa*	ATCC	.	>800	>800	>800	>800	>800	>800	>800	>800	>800
*E. coli*	ATCC	>800	>800	>800	>800	>800	>800	>800	>800	>800	>800
*S. enterica*	204/22	>800	>800	>800	>800	>800	>800	>800	>800	>800	>800
*P. multocida*	380/22	50	>800	50	400	50	400	>800	800	800	800
*S. aureus*	ATCC	50	>800	100	800	100	800	200	800	800	800
*S. suis*	672/22	<1.5625	>800	3.125	50	6.25	50	50	400	200	200
*B. cepacia*	20-10299	800	>800	400	800	400	800	800	800	800	400
*E. fecalis*	442	>800	>800	>800	>800	>800	>800	>800	>800	>800	>800

## Data Availability

All raw data supporting the results of the present study can be achieved from the corresponding author upon reasonable request.

## Supplementary Materials

The following supporting information can be downloaded at: https://www.mdpi.com/article/10.3390/biomedicines12040783/s1, Figure S1: Matriptase/TMPRSS2 inhibitors applied in this study besides MI-432, MI-471, and MI-476 [32,56]. The chemical structures of the additionally used inhibitors for MIC studies are provided in Figure S1.
