# Supplementary material for: Antiviral Drug Candidate Repositioning for Streptococcus suis Infection in Non-Tumorigenic Cell Models
Source: Biomedicines. 2024 Apr 2;12(4):783. doi: 10.3390/biomedicines12040783 (PMC11048155; doi:10.3390/biomedicines12040783)
Supplement: Supplementary file 1 [file biomedicines-12-00783-s001.zip › biomedicines-2915239-supplementary.pdf]

Supplementary material

MI-463

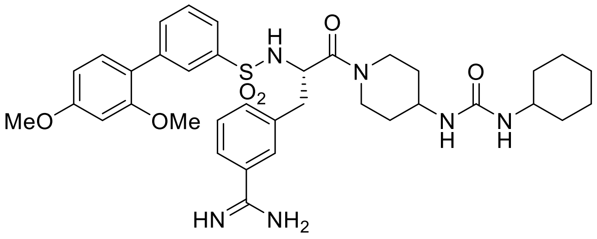

MI-472

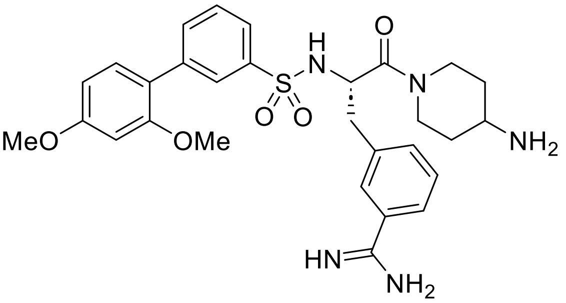

MI-477

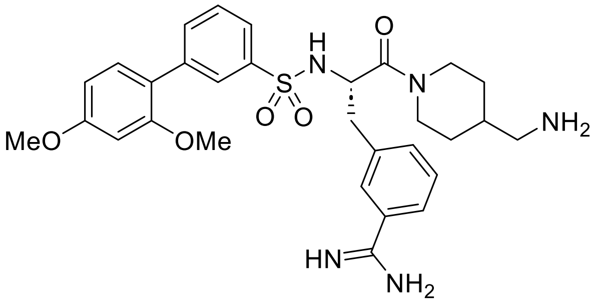

MI-485

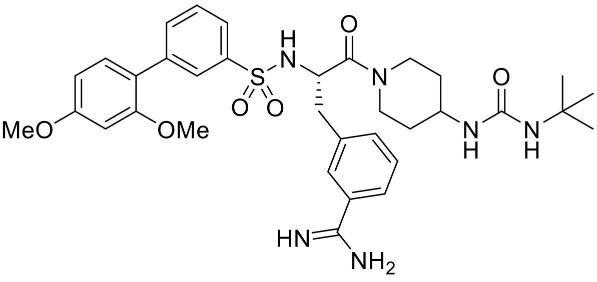

MI-490

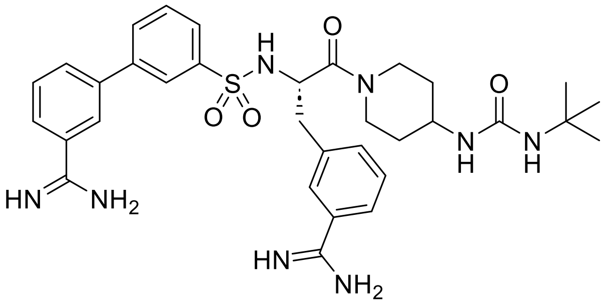

MI-1903

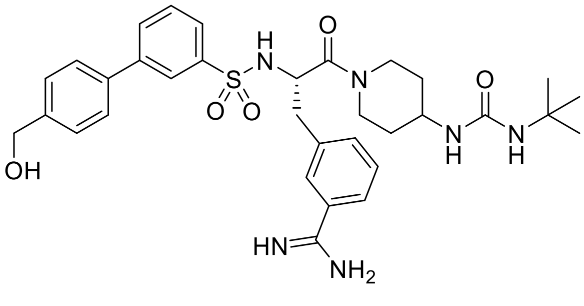

MI-1904

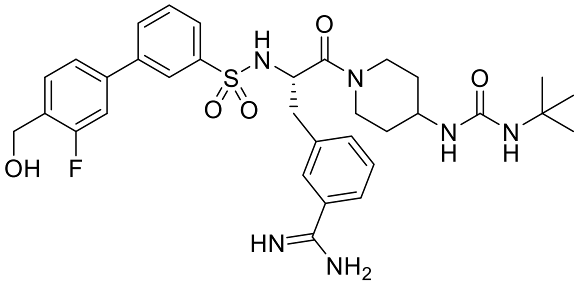

Figure S1. Matriptase/TMPRSS2 inhibitors applied in this study besides MI-432, MI-471 and MI-476 [32,56].
